# Acupuncture for breathlessness in COVID-19: A protocol for systematic review and meta-analysis

**DOI:** 10.1097/MD.0000000000020701

**Published:** 2020-07-02

**Authors:** Baozhen Zhang, Kai Zhang, Qilin Tang, Kaihang Sun, Zhenzhen Han

**Affiliations:** a Department of Health Management, Tianjin Rehabilitation and Convalescence Center of PLA; b Department of Acupuncture and Moxibustion, Tianjin Gong An Hospital, Tianjin; c School of Basic Medical Sciences, Hebei University of Chinese Medicine, Hebei, Shijiazhuang; d Department of Acupuncture and Moxibustion, First Teaching Hospital of Tianjin University of Traditional Chinese Medicine; e National Clinical Research Center for Chinese Medicine Acupuncture and Moxibustion, Tianjin, China.

**Keywords:** acupuncture, COVID-19, protocol, randomized controlled trials, systematic review

## Abstract

**Background:**

At present, accumulative attention has been paid to coronavirus disease 2019 (COVID-19) due to its global prevalence. Acupuncture may play a beneficial role in patients with breathlessness in COVID-19. This study is designed to determine the efficacy and safety of acupuncture for breathlessness in COVID-19.

**Methods:**

Randomized controlled trials (RCT) will be searched from 7 electronic databases, with the last search update being 30 June 2020. Studies by registers of clinical trials will be additionally searched. Two investigators will independently select studies, extract data and evaluate study quality. Finally, a meta-analysis will be used to evaluate the pooled intervention effect if possible.

**Results:**

Our present findings will indicate the application of acupuncture as an adjunctive treatment for dyspnea in COVID-19, which will be published in a peer-reviewed journal.

**Conclusion:**

Our study will provide a reference foundation for clinical optimization of treatment.

**Prospero registration number:**

CRD42020182323.

## 1 Introduction

Coronaviruses (CoVs) is a type of enveloped positive-sense RNA virus that is diversely found in humans and wildlife.^[1]^ Human coronaviruses (HCoVs) have been proven to be non-essential pathogens for a long period, which can result in “common cold” in other healthy population.^[1,2]^ Nevertheless, two highly pathogenic HCoVs from animal reservoirs have led to worldwide pandemics with striking morbidity and mortality, including severe acute respiratory syndrome coronavirus (SARS-CoV) and Middle East respiratory syndrome coronavirus (MERS-CoV).^[2]^ At present, a new strain called the severe acute respiratory syndrome coronavirus 2 (SARS-CoV-2) virus has become another outbreak. The coronavirus disease 2019 (COVID-19) was recognized in Wuhan, China in December, 2019, causing increasing morbidity and mortality ever since.^[2]^ The ongoing COVID-19 pandemic is an exceptional challenge for the health systems throughout the world. Through the continuous accumulation of research data, the biological, epidemiological, and clinical characteristics of COVID -19 have been gradually improved. International attention has been paid to COVID-19 due to the quickly increasing number of diagnosed patients along with subsequently rising secondary outbreaks in various global regions. Hopefully, the rapid in-depth sequencing of viral genomes has enabled the development and research of diagnostic tests, as well as the initiation of vaccine and therapeutics research.^[3]^ However, so far, no effective vaccine or causative therapy is available.^[3]^ Existing studies have readily confirmed the interpersonal transmission of SARS-CoV-2,^[3,4]^ with an incubation period varying from 1 to 14 days (median: 5–6 days), which could be 24 days under extreme conditions.^[3,5]^ Additionally, patients with COVID-19 have a higher proportion of men, the elderly, patients with hypertension and/or diabetes.^[6]^ There are also investigations concerning the risk factors as well as clinical outcomes in admission and intensive care unit. In an early study of patients with COVID-19 in China and Italian, older men, patients with smoking and cardiometabolic diseases were associated with poor prognosis.^[7]^ In general, the clinical presentation of COVID-19 includes myalgias, fatigue dry cough and fever, as well as less common ones (including abdominal pain, breathlessness, headache diarrhea and sore throat).^[3,8]^ A recent descriptive study found that there were 56.7% men among elderly diagnosed COVID-19 patients were male, and common symptoms included fatigue (23.3%), dyspnea (30.0%), cough (56.7%), and fever (78.3%).^[8]^ In general, older population are more vulnerable to COVID-19 infection, and their mortality rate is higher, which deserves more attention.^[8]^ The COVID-19 pandemic has triggered prevalent research interest in therapeutic and preventive interventions. However, due to the lack of specific antiviral therapeutics and vaccine, adjuvant therapy has become the major therapeutic strategy for COVID-19, along with the administration of corticosteroids, antivirals, broad-spectrum antibiotics and convalescent plasma.^[9]^ At present, diverse trials have been launched, such as tocilizumab, losartan, hydroxychloroquine, remdesivir as well as convalescent plasma.^[10]^

**Figure 1:**
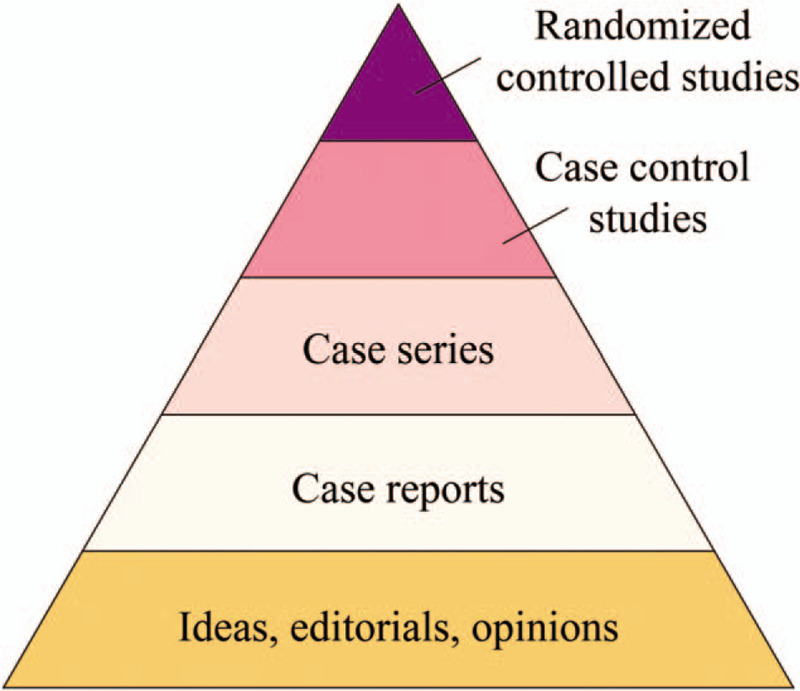
Types of current evidence on TCM treatment of COVID-19.

Traditional Chinese medicine (TCM) plays an important role in the prevention and treatment of various infectious diseases. To be specific, the application of TCM has also obtained significant therapeutic efficacy during the SARS epidemic in 2003.^[11]^ The combination of TCM and Western medicines could relieve symptoms, enhance life quality, absorb lung infiltration, while attenuate the corticosteroid dosage among SARS patients.^[11]^ Similarly, recent clinical practice has also revealed the significant therapeutic effect of TCM in COVID-19.^[12]^ China has issued guidelines for the diagnosis and treatment of COVID-19, recommending the use of conventional treatment methods plus TCM.^[9,13]^ In the current Chinese medical system, licensed practitioners of TCM are allowed to prescribe western medicines after a formal course of study.^[14]^ Therefore, the participation of TCM practitioners in the treatment of COVID-19 is legal and supported by the government. During the entire treatment period of COVID-19, Chinese medical staff volunteered to join the designated hospitals in Hubei Province,^[12]^ who comprehensively adopted acupuncture, Chinese patent medicine, decoction and other characteristic therapies of TCM, shedding novel light on the control and prevention of COVID-19.^[12]^ In total, 303 ongoing clinical trials concerning the assessment of the therapeutic safety and efficacy for COVID-19 patients have been launched in China by March 1, 2020, 50 of which focus on TCM, including 14 clinical trials aimed at evaluating the efficacy of TCM combined with Western medicine.^[15]^ At present, there are various types of evidence for TCM treatment of COVID-19,^[9,13,15]^ as shown in Figure 1.

**Figure 2:**
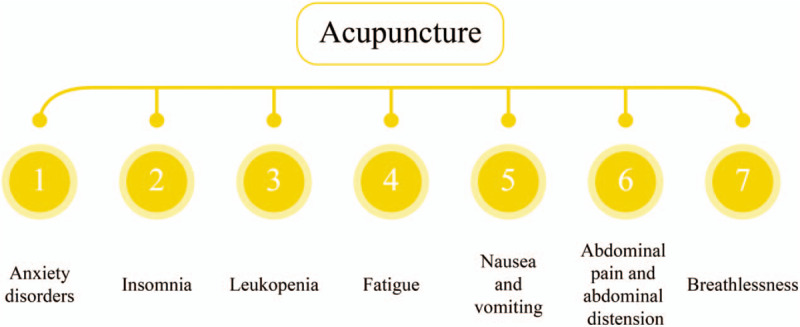
Possible related symptoms of COVID-19 treated with acupuncture.

Acupuncture, a main component of TCM, has been widely adopted to treat respiratory diseases in clinical practice,^[16,17]^ whose efficacy has been assessed by a number of randomized controlled trials (RTCs).^[18]^ Breathlessness is one of the prevalent symptoms in COVID-19 patients.^[8,19]^ Acupuncture may play a role in the prevention, treatment and rehabilitation of the COVID-19 and relieve the symptoms caused by COVID-19. Acupuncture has been demonstrated to effectively relieving common symptoms in supportive and palliative care, including anxiety disorders, nausea, insomnia, leukopenia, fatigue as well as vomiting,^[20–25]^ which might also effectively treat abdominal pain and abdominal distension.^[26,27]^ Coyle et al have proposed that acupuncture is an effective therapeutic approach for COPD-associated breathlessness.^[28]^ Possible related symptoms of COVID-19 treated with acupuncture is shown as Figure 2. The recent systematic review and meta-analysis show that acupuncture can relieve breathlessness in subjects with advanced diseases.^[16]^ Therefore, in this meta-analysis review, our goal is to systematically review the efficacy of acupuncture in relieving breathlessness, subsequently improving the physiological function and quality of life of patients with COVID-19 combined with dyspnea.

## 2 Methods

The study will be conducted in accordance with the preferred reporting items for systematic review and meta-analysis protocols (PRISMA-P).^[29]^

### 2.1 Inclusion criteria

#### 2.1.1 Study type

Randomized controlled trials (RCTs) will be included in the review, without restriction on language or publication date.

#### 2.1.2 Participant types

Patients with breathlessness due to lab-confirmed COVID-19 will be included, regardless of age, race, sex. Diagnosis of COVID-19 is based on the international or Chinese diagnostic criteria for COVID-19.^[3,30,31]^

#### 2.1.3 Types of interventions

Acupuncture will be performed in the treatment group, combined with other treatments, including routine therapy and so on.^[9]^ Patients in the control group will receive other therapeutic approaches other than acupuncture, including routine therapy, placebo, etc.

#### 2.1.4 Types of outcomes

The primary outcomes include the changes on any subjective measurement of breathlessness severity made on a validated rating scale from baseline to endpoint, including visual analogue scale, numerical rating scale and the Borg Scale.^[32,33]^

Secondary outcomes include the assessment of quality of life activities using any validated questionnaire. In addition, anxiety will be evaluated by any validated scale. The safety of treatment will be assessed by the incidence and degree of adverse events.^[34–36]^

### 2.2 Search methods to identify studies

#### 2.2.1 Search strategy

Two reviewers will independently search seven databases: EMBASE, Medline, Cochrane Central Register of Controlled Trials, Web of Science, Wan Fang Data, China National Knowledge Infrastructure as well as Chinese Scientific Journal Database (VIP) from inception until 30 June 2020 (search date). The same terms in China will be conducted in Chinese databases. The searching strategy for Medline is shown in Table 1.

**Table 1:**
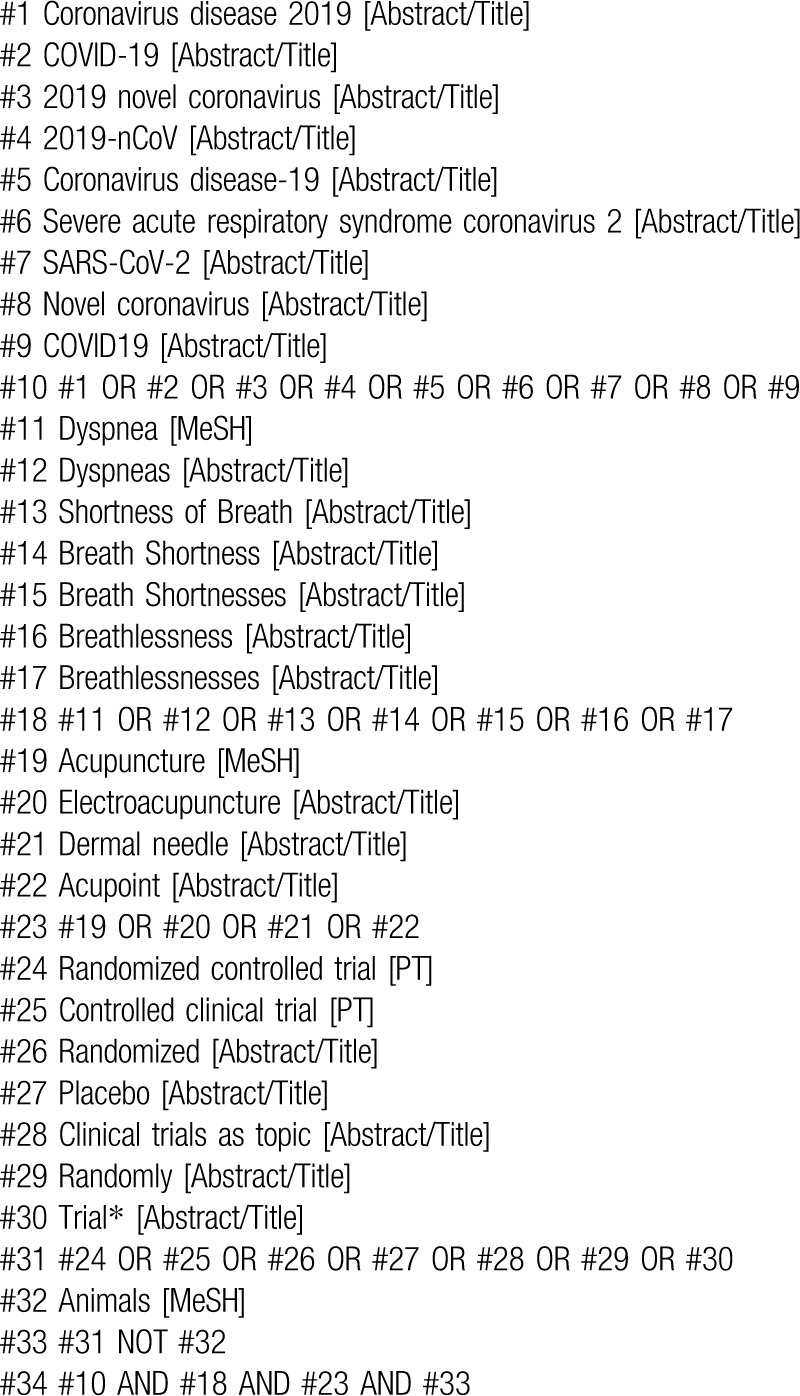
Search strategy for Medline.

#### 2.2.2 Searching other resources

Similar retrieval methods will be applied in the Clinical Trials.gov to obtain unpublished studies. There is no restriction on publication regions or language.

### 2.3 Data collection and analysis

#### 2.3.1 Selection of studies

**Figure 3:**
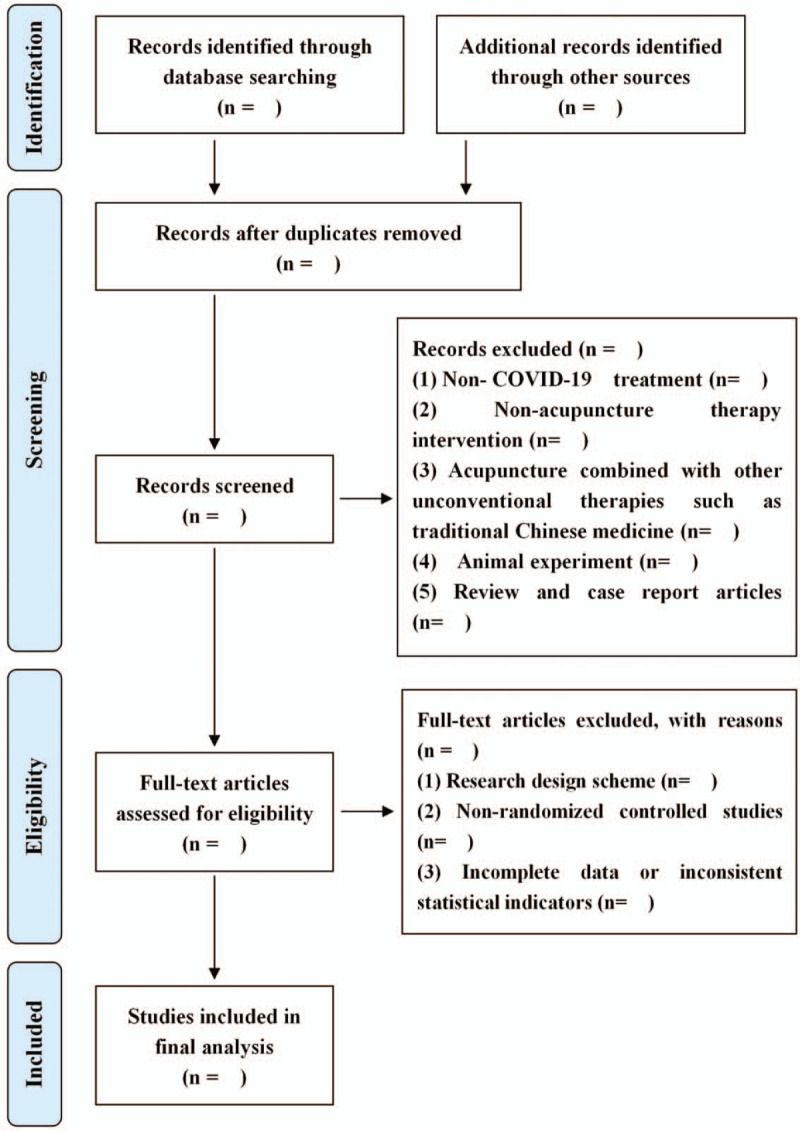
Study selection flow diagram.

After searching studies, 2 investigators will review titles and abstracts, or full text if necessary. All investigators will reach agreement on the identification of study inclusion after evaluating their eligibility, and we will also record reasons why studies are eliminated. The selection process is summarized using PRISMA flow diagram. Details of the selection procedure for studies are shown in a PRISMA flow chart (Fig. 3). Any inconsistency is resolved by discussing with a third investigator.

#### 2.3.2 Data extraction and management

Two investigators will extract relevant data independently, including study design, general information, characteristics of patients, comparison interventions, and outcomes. For articles with incomplete or uncertain data, the authors will be contacted for complete data. And the study will be further excluded without adequate information. If there is any dispute in the data extraction process, it will be submitted to a third researcher for processing.

#### 2.3.3 Evaluation of bias risk in included studies

Two investigators will independently evaluate the bias risks among enrolled researches according to the Cochrane Collaboration.^[37]^ Discrepancy will be resolved by discussion and judgment by an arbiter. The following seven aspects in all RCTs will be evaluated: outcome assessment blinding, participants and personnel blinding, selective outcome reporting, allocation concealment, inadequate outcome data, generation of random sequences as well as other potential sources of bias.^[37]^ The bias risk in each aspect will be assessed and divided into 3 levels: low risk, high risk, and unclear risk.

#### 2.3.4 Measurement of therapeutic effect

Review Manager (RevMan 5.3) software will be used for statistical analysis if a meta-analysis is allowed. Risk ratio will be calculated for dichotomous data. The intervention effect will be shown as the mean difference for continuous outcomes. Additionally, 95% confidence intervals will be calculated.

#### 2.3.5 Heterogeneity evaluation

After stratifying the study according to the therapeutic duration and region, the chi-square test will be utilized to evaluate the heterogeneity and depressive symptoms. I^2^ statistic will be utilized for quantification of heterogeneity degree, where I^2^ >50% indicates the significant heterogeneity.^[37–40]^

#### 2.3.6 Publication bias

The publication bias will be evaluated by funnel plots by determining whether there are 10 or more studies with the same outcome. In the case of asymmetric funnel plot, subgroup analysis or sensitivity analysis will be performed to investigate possible causes.^[37,41]^

#### 2.3.7 Data synthesis

RevMan 5.3 software will be utilized for statistical analysis. The fixed-effects model will be employed to analyze data in the case of insignificant heterogeneity (I^2^ < 50%). In the case of heterogeneity (I^2^ ≥ 50%), subgroup analysis will be further conducted to decrease the clinical heterogeneity by taking into consideration of possible factors. If the heterogeneity is still significant, the random-effect model or qualitative description will be used.

#### 2.3.8 Sensitivity analysis

Sensitivity analysis will be conducted by sequential omission of single study at a time, followed by simultaneously excluding 2 studies for identification of factors making the most contribution to heterogeneity.

#### 2.3.9 Assessment of evidence quality

The Grading of Recommendations Assessment, Development and Evaluation (GRADE) evaluation method of evidence quality will be used to evaluate the primary and secondary results of this study. The evidence quality will be categorized into high, moderate, low or very low according to five parameters (publication bias, indirectness, inconsistency, imprecision, and study limitations).

### 2.4 Ethics and publication

Since this study does not involve the patient privacy, ethical approval is not required. Our research results will be shared and shown through conference reports and peer-reviewed journals.

## 3 Discussion

The pathogenesis and clinical symptoms related to severe respiratory disease were described many years ago in TCM texts.^[42]^ There are many studies on current application of TCM in COVID-19,^[42,43]^ such as the clinical outcome, pathogenesis and the current application of TCM on COVID-19. The strength of our review includes the following 3 points. First, it is the first systematic review concerning the safety and effectiveness of acupuncture for breathlessness in COVID-19. Second, only RCTs are included in our systematic review, which are more likely to provide unbiased information than other study designs. Third, the comprehensive search strategy renders in-depth searching lists as well as trial registries associated with acupuncture and COVID-19.

However, the intrinsic methodological challenges among these enrolled trials will limit our systematic review. As a manipulated intervention, it is difficult to implement the blindness of therapeutic modes on acupuncturists.^[44–48]^ Acupuncture therapy could be further categorized into manipulation and needling instrument. In addition, there might be great variation on acupuncture therapy in these enrolled studies. Although the above problem might be resolved and the consistency of interventions might be ensured by subgroup analysis, the comparability of enrolled researches will be decreased and the difficulty in meta-analysis will be increased.

## Author contributions

**Conceptualization:** Baozhen Zhang and Kai Zhang.

**Data curation:** Kai Zhang and Zhenzhen Han.

**Formal analysis:** Kai Zhang and Zhenzhen Han.

**Investigation:** Baozhen Zhang and Kai Zhang.

**Methodology:** Baozhen Zhang.

**Software:** Qilin Tang and Kaihang Sun.

**Supervision:** Qilin Tang.

**Writing – original draft:** Baozhen Zhang, Kai Zhang, and Kaihang Sun.

**Writing – review & editing:** Qilin Tang.

## References

[1] Fehr AR Perlman S Coronaviruses: an overview of their replication and pathogenesis. Methods Mol Biol 2015;1282:1–23. 25720466 10.1007/978-1-4939-2438-7_1 PMC4369385

[2] Paules CI Marston HD Fauci AS Coronavirus Infections-More Than Just the Common Cold. JAMA 2020. 10.1001/jama.2020.0757 31971553

[3] Del Rio C Malani PN COVID-19-new insights on a rapidly changing epidemic. JAMA 2020. 10.1001/jama.2020.3072 32108857

[4] Wang C Horby PW Hayden FG A novel coronavirus outbreak of global health concern. Lancet 2020;395:470–3. 31986257 10.1016/S0140-6736(20)30185-9 PMC7135038

[5] Bai Y Yao L Wei T Presumed asymptomatic carrier transmission of COVID-19. JAMA 2020. 10.1001/jama.2020.2565 PMC7042844 32083643

[6] Zhou F Yu T Du R Clinical course and risk factors for mortality of adult inpatients with COVID-19 in Wuhan, China: a retrospective cohort study. Lancet 2020;395:1054–62. 32171076 10.1016/S0140-6736(20)30566-3 PMC7270627

[7] Wu Z McGoogan JM Characteristics of and important lessons from the coronavirus disease 2019 (COVID-19) outbreak in China: summary of a report of 72 314 cases from the Chinese center for disease control and prevention. JAMA 2020;323:1239–42. 10.1001/jama.2020.2648 32091533

[8] Niu S Tian S Lou J Clinical characteristics of older patients infected with COVID-19: a descriptive study. Arch Gerontol Geriatr 2020;89:104058. 32339960 10.1016/j.archger.2020.104058 PMC7194515

[9] Jin YH Cai L Cheng ZS A rapid advice guideline for the diagnosis and treatment of 2019 novel coronavirus (2019-nCoV) infected pneumonia (standard version). Mil Med Res 2020;7:4. 32029004 10.1186/s40779-020-0233-6 PMC7003341

[10] Dong L Hu S Gao J Discovering drugs to treat coronavirus disease 2019 (COVID-19). Drug Discov Ther 2020;14:58–60. 32147628 10.5582/ddt.2020.01012

[11] Liu X Zhang M He L Chinese herbs combined with Western medicine for severe acute respiratory syndrome (SARS). Cochrane Database Syst Rev 2012;10:CD004882. 23076910 10.1002/14651858.CD004882.pub3 PMC6993561

[12] Ren JL Zhang AH Wang XJ Traditional Chinese medicine for COVID-19 treatment. Pharmacol Res 2020;155:104743. 32145402 10.1016/j.phrs.2020.104743 PMC7128263

[13] Zhang K Is traditional Chinese medicine useful in the treatment of COVID-19? Am J Emerg Med 2020. 10.1016/j.ajem.2020.03.046 PMC7138187 32245701

[14] Zhang K Tang Q The dilemma and hope of Traditional Chinese Medicine practitioners in China. Integr Med Res 2020. 10.1016/j.imr.2020.100411 PMC7193313 32373462

[15] Yang Y Islam MS Wang J Traditional Chinese medicine in the treatment of patients infected with 2019-new coronavirus (SARS-CoV-2): a review and perspective. Int J Biol Sci 2020;16:1708–17. 32226288 10.7150/ijbs.45538 PMC7098036

[16] von Trott P Oei SL Ramsenthaler C Acupuncture for breathlessness in advanced diseases: a systematic review and meta-analysis. J Pain Symptom Manage 2020;59:327–38. e3. 31539602 10.1016/j.jpainsymman.2019.09.007

[17] Zhang K Li Y Tang Q Acupuncture for breathlessness in advanced diseases: methodological issues. J Pain Symptom Manage 2020;59:e3–4. 10.1016/j.jpainsymman.2019.11.020 31805365

[18] Kaptchuk TJ Acupuncture: theory, efficacy, and practice. Ann Intern Med March 2002;136:374–83. 10.7326/0003-4819-136-5-200203050-00010 11874310

[19] Lovell N Maddocks M Etkind SN Characteristics,symptom management and outcomes of 101 patients with COVID-19 referred for hospital palliative care. J Pain Symptom Manage 2020. 10.1016/j.jpainsymman.2020.04.015 PMC7169932 32325167

[20] Cheong KB Zhang JP Huang Y The effectiveness of acupuncture in prevention and treatment of postoperative nausea and vomiting--a systematic review and meta-analysis. PLoS One 2013;8:e82474. 24349293 10.1371/journal.pone.0082474 PMC3862842

[21] Zhang Y Lin L Li H Effects of acupuncture on cancer-related fatigue: a meta-analysis. Support Care Cancer 2018;26:415–25. 29128952 10.1007/s00520-017-3955-6

[22] Tang Q Wang S Zhang K Interventions for sleep problems during pregnancy: a systematic review. Sleep Med Rev 2020;51:101287. 32120166 10.1016/j.smrv.2020.101287

[23] Lee SH Lim SM Acupuncture for insomnia after stroke: a systematic review and meta-analysis. BMC Complement Altern Med 2016;16:228. 27430619 10.1186/s12906-016-1220-z PMC4950252

[24] Amorim D Amado J Brito I Acupuncture and electroacupuncture for anxiety disorders: a systematic review of the clinical research. Complement Ther Clin Pract 2018;31:31–7. 29705474 10.1016/j.ctcp.2018.01.008

[25] Tang Q Zhang K Association of acupuncture and acupressure with improved cancer pain. JAMA Oncol 2020. 10.1001/jamaoncol.2020.0616 32324212

[26] Zhang K Gao C Li C Acupuncture for acute pancreatitis: a systematic review and meta-analysis. Pancreas 2019;48:1136–47. 31593017 10.1097/MPA.0000000000001399

[27] Zhang K Li C Gao C Efficacy and safety of acupuncture as an adjuvant treatment for acute pancreatitis: a protocol of systematic review and meta-analysis. BMJ Open 2019;9:e029327. 10.1136/bmjopen-2019-029327 PMC6615812 31278104

[28] Coyle ME Shergis JL Huang ET Acupuncture therapies for chronic obstructive pulmonary disease: a systematic review of randomized, controlled trials. Altern Ther Health Med 2014;20:10–23. 25478799

[29] Shamseer L Moher D Clarke M Preferred reporting items for systematic review and meta-analysis protocols (PRISMA-P) 2015: elaboration and explanation. BMJ 2015;349:g7647. 10.1136/bmj.g7647 25555855

[30] National Health Commission of the People's Republic of China.<Guideline on diagnosis and treatment of COVID-19 (Trial 6th edition). http://www.nhc.gov.cn/xcs/zhengcwj/202002/8334a8326dd94d329df351d7da8aefc2.shtml. [access date February 23, 2020].

[31] Ahn DG Shin HJ Kim MH Current status of epidemiology, diagnosis, therapeutics, and vaccines for novel coronavirus disease 2019 (COVID-19). J Microbiol Biotechnol 2020;30:313–24. 32238757 10.4014/jmb.2003.03011 PMC9728410

[32] Aitken RC Measurement of feelings using visual analogue scales. Proc R Soc Med 1969;62:989–93. 4899510 10.1177/003591576906201005 PMC1810824

[33] Borg GA Psychophysical bases of perceived exertion. Med Sci Sports Exerc 1982;14:377–81. 7154893

[34] Zhang K Li Y Tang Q Acupuncture for stable angina pectoris: A few noteworthy additions. Eur J Prev Cardiol 2019. 10.1177/2047487319889721 31744336

[35] Wang Q Tang Q Zhang K Letter to the editor regarding “acupuncture-induced cranial epidural abscess: case report and review of the literature”. World Neurosurg 2019;132:443. 31810147 10.1016/j.wneu.2019.07.174

[36] Tang Q Tian L Gao C The efficacy and safety of Xuebijing injection as an adjunctive treatment for acute pancreatitis: Protocol for a systematic review and meta-analysis of randomized controlled trials. Medicine (Baltimore) 2020;99:e18743. 31977866 10.1097/MD.0000000000018743 PMC7004790

[37] Higgins JPT Green S Cochrane handbook for systematic reviews of interventions version 5.1.0 [updated March 2011]. The Cochrane Collaboration, 2011. http://www.handbook.cochrane.org.

[38] Zhang K Is Nigella Sativa Supplementation Effective for Asthma? Am J Emerg Med 2020. 10.1016/j.ajem.2020.02.007 32063428

[39] Tang Q Zhang K Is endoscopic retrograde cholangiopancreatography safe during pregnancy? Saudi J Gastroenterol 2020;26:61–2. 31997780 10.4103/sjg.SJG_624_19 PMC7045774

[40] Zhang K Water exchange versus air insufflation for colonoscopy: methodological issues of the meta-analysis are a cause for concern. Saudi J Gastroenterol 2019;25:205. 31044744 10.4103/sjg.SJG_42_19 PMC6526740

[41] Zhang K Xuebijing combined with ulinastation for sepsis:a few noteworthy additions. Am J Emerg Med 2020. 10.1016/j.ajem.2020.02.008 32067839

[42] Li M Yang X Li K Traditional Chinese medicine for novel coronavirus pneumonia treatment: main force or supplement? Trad Med Res 2020;5:62–4.

[43] Cui HT Li YT Guo TY Traditional Chinese medicine for treatment of coronavirus disease 2019: a review. Trad Med Res 2020;5:65–73.

[44] Zhang K Tang Q Acupuncture on aromatase inhibitor-induced arthralgia in patients with breast cancer. Breast 2019;45:119. 10.1016/j.breast.2019.01.010 31029376

[45] Zhang K Gao C Tang Q Acupuncture for reduction of symptom burden in multiple myeloma patients undergoing autologous hematopoietic stem cell transplantation: a randomized sham-controlled trial. Respond to author. Support Care Cancer 2019;27:3171–2. 30868268 10.1007/s00520-019-04732-1

[46] Tang Q Zhang K Is acupuncture effective for knee osteoarthritis? Comment on a recent trial. Clin Rehabil 2019;33:1697–8. 31304786 10.1177/0269215519863554

[47] Zhang K Tang Q Zhao C Traditional manual acupuncture combined with rehabilitation therapy for shoulder hand syndrome after stroke within the Chinese healthcare system. Clin Rehabil 2019;33:1699–700. 31519132 10.1177/0269215519877739

[48] Zhang K Tang Q Gao C Non-pharmacologic treatments for symptoms of diabetic peripheral neuropathy: a systematic review-methodological issues are a matter for concern. Curr Med Res Opin 2019;35:1319–20. 30892099 10.1080/03007995.2019.1598135

